# Gut Microbiota Confers Resistance of Albino Oxford Rats to the Induction of Experimental Autoimmune Encephalomyelitis

**DOI:** 10.3389/fimmu.2018.00942

**Published:** 2018-05-02

**Authors:** Suzana Stanisavljević, Miroslav Dinić, Bojan Jevtić, Neda Đedović, Miljana Momčilović, Jelena Đokić, Nataša Golić, Marija Mostarica Stojković, Đorđe Miljković

**Affiliations:** ^1^Department of Immunology, Institute for Biological Research “Siniša Stanković”, University of Belgrade, Belgrade, Serbia; ^2^Laboratory for Molecular Microbiology, Institute of Molecular Genetics and Genetic Engineering, University of Belgrade, Belgrade, Serbia; ^3^School of Medicine, Institute for Microbiology and Immunology, University of Belgrade, Belgrade, Serbia

**Keywords:** experimental autoimmune encephalomyelitis, multiple sclerosis, gut microbiota, gut-associated lymphoid tissue, antibiotics, gut microbiota transfer

## Abstract

Albino Oxford (AO) rats are extremely resistant to induction of experimental autoimmune encephalomyelitis (EAE). EAE is an animal model of multiple sclerosis, a chronic inflammatory disease of the central nervous system (CNS), with established autoimmune pathogenesis. The autoimmune response against the antigens of the CNS is initiated in the peripheral lymphoid tissues after immunization of AO rats with CNS antigens. Subsequently, limited infiltration of the CNS occurs, yet without clinical sequels. It has recently become increasingly appreciated that gut-associated lymphoid tissues (GALT) and gut microbiota play an important role in regulation and propagation of encephalitogenic immune response. Therefore, modulation of AO gut microbiota by antibiotics was performed in this study. The treatment altered composition of gut microbiota in AO rats and led to a reduction in the proportion of regulatory T cells in Peyer’s patches, mesenteric lymph nodes, and in lymph nodes draining the site of immunization. Upregulation of interferon-γ and interleukin (IL)-17 production was observed in the draining lymph nodes. The treatment led to clinically manifested EAE in AO rats with more numerous infiltrates and higher production of IL-17 observed in the CNS. Importantly, transfer of AO gut microbiota into EAE-prone Dark Agouti rats ameliorated the disease. These results clearly imply that gut microbiota is an important factor in AO rat resistance to EAE and that gut microbiota transfer is an efficacious way to treat CNS autoimmunity. These findings also support the idea that gut microbiota modulation has a potential as a future treatment of multiple sclerosis.

## Introduction

The origin of adaptive immune system has been associated with establishment of a commensal relation of vertebrates and gut microorganisms (1). Also, it is becoming increasingly appreciated that the immune system is deeply influenced by the gut microorganisms (2). Therefore, it does not come as a surprise that autoimmunity, as one of the major disorders arising from inappropriate activity of the adaptive immune system, has been tightly connected to gut microbiota perturbations (3). Multiple sclerosis is a chronic inflammatory disease of the central nervous system (CNS). While autoimmune response directed against the CNS has been considered as the major element of the disease pathogenesis, the initial steps of the autoimmunity have still remained elusive (4). Based on data obtained from studies on animal models of multiple sclerosis the role of gut microbiota in the initiation of the CNS autoimmunity has been suggested (4). The primary step in the autoimmune process is activation of the CNS-reactive autoimmune CD4^+^ T cells (T helper, Th cells) that differentiate toward interferon (IFN)-γ-producing Th1 cells and interleukin (IL)-17-producing Th17 cells (5). Molecular mimicry between gut microbes and the CNS antigens has been suggested as a possible scenario for activation and differentiation of the CNS autoreactive Th1 and Th17 cells within the GALT (4, 6). Although direct evidences on the role of gut microbiota in the autoimmune pathogenesis of multiple sclerosis are still missing, there are correlative studies that show that intestinal microbiota is changed, presumably dysbiotic in multiple sclerosis patients. For instance, lower abundance of *Faecalibacterium* (7), clostridia clusters XIVa, IV, *Bacteroides fragilis, Sutterella wadsworthensis* (8), *Butyricimonas* (9), *Parabacteroides, Adlercreutzia*, and *Prevotella* operational taxonomic units (10) was detected in multiple sclerosis patients. In contrast, *Methanobrevibacter* and *Akkermansia* (9), *Pseudomonas, Mycoplana, Haemophilus, Blautia*, and *Dorea* genera (10) were increased in the patients. Recently published reports reveal new bacterial genera over presented in multiple sclerosis microbiome such as *Acinetobacter* (11). Moreover, evidence for the functional significance of altered multiple sclerosis microbiota was provided by its transfer into germ free mice which resulted in potentiating experimental autoimmune encephalomyelitis (EAE) clinical signs (11). Alike, higher incidence of CNS autoimmunity in spontaneous model of EAE after colonization of germ-free RR mice with microbiota from multiple sclerosis affected twins compared to that obtained from healthy twins (12). Discovery of multiple sclerosis-related pathogenic and/or protective gut microbiota organisms or their products would open novel opportunities for diagnosis and therapy of the disease. The latter would rely on antibiotic (Antb) and/or probiotic treatments, as well as on gut microbiota transfer from healthy donors. However, it is already known that gut microbiota is individually tailored and that it is hard to efficiently change composition of gut microbiota (13). Thus, further studies with experimental models are needed.

Our primary aim was to explore whether perturbation of gut microbiota reflects on susceptibility to induction of EAE, an animal model of multiple sclerosis. Albino Oxford (AO) rats resistant to induction of EAE and Dark Agouti (DA) rats prone to the disease (14) were used in this study. We have recently shown that these two rat strains differ in composition of their gut microbiota (15, 16). Higher diversity of bacteria was detected in DA rats. Specifically, in most of AO rat samples only lactobacilli and enterococci were detected, while DA rat samples contained other bacteria, including Lachnospiraceae, Firmicutes, and Proteobacteria (Burkholderiales, *Undibacterium oligocarboniphilum*) (15, 16). Moreover, AO gut microbiota did not change considerably after EAE immunization, while extensive changes were demonstrated in DA rats after the immunization. Therefore, it was plausible to assume that AO gut microbiota contributed to their resistance to EAE induction. Antb treatment was used in our experiments to disturb AO gut microbiota and to overcome their resistance to EAE induction. Further, our goal was to test if gut microbiota transfer from AO rats to EAE-prone DA rats would change DA gut microbiota composition and consequently protect them from the disease. Cecal content was grafted in our experiments as it was a reliable source of gut microorganisms.

## Materials and Methods

### Experimental Animals and EAE

Female AO and DA rats were bred and maintained in the animal facility of the Institute for Biological Research “SiniŠa StankoviĆ.” Animal experiments were approved by the local ethics committee (Institute for Biological Research “SiniŠa StankoviĆ,” No. 04-04/15). Housing of the rats was performed under the same environmental conditions. Three to five rats were kept in the same cage. EAE was induced with DA rat spinal cord homogenate (SCH) in phosphate buffer saline (PBS, 50% w/v) mixed with equal volume of complete Freund’s adjuvant (CFA, Difco, Detroit, MI, USA) supplemented with 5 mg/ml of *M. tuberculosis* H37Ra (Difco). Alternatively, rats were immunized with myelin basic protein (guinea pig MBP, 50 μg/rat, kind gift from Professor Alexander Flügel, University of Göttingen, Germany), emulsified with equal volume of CFA supplemented with 5 mg/ml of *M. tuberculosis* H37Ra. MBP + CFA immunization was performed for subsequent antigen-specific production of cytokines that was determined in cells of lymph nodes draining the site of injection [popliteal lymph nodes (PLN) were extracted from the rats on day 4 and day 7 after the immunization]. The animals were injected subcutaneously into hind limbs with 100 µl of either emulsion. The rats were monitored daily for clinical score (c.s.) of EAE, and scored according to the following scale: 0, no clinical signs; 1, flaccid tail; 2, hind limb paresis; 3, hind limb paralysis; 4, moribund state or death. Cumulative c.s. was calculated as sum of daily c.s. Duration was number of days that the clinical signs were observed in each of the rats. Mean c.s. was calculated as cumulative c.s. divided by duration. In order to detect inflammatory infiltrates in the CNS of immunized animals, hematoxylin and eosin staining was performed. The stained cells were counted from five animals. Five micrometers of transversal lumbosacral sections (L1–L5) were cut and eight successive slices were placed on the same glass slide. Number of infiltrates and cells per infiltrate were counted from histological data obtained from 120 sections per group.

### Antb Treatment

Antibiotic treatment consisted of 2.5 g/l of neosulfox [Sulfadimidine sodium 10% (w/w), neomycin sulfate 6%, oxytetracycline hydrochloride4%, “Fm Pharm” d.o.o., Subotica], 2.5 g/l pentrexyl (ampicillin, Galenika, Belgrade, Serbia). The Antb were applied to AO rats in drinking water and changed regularly every second day. The treatment started at the day of birth and was introduced to rats gradually from 0.5 to 2.5 g/l. The full dosage was achieved at day 7 *postpartum*. Rats were allowed to drink the water with Antb *ad libitum* for 4 weeks, i.e., throughout the duration of the preweaning period. Then, rats were separated from mothers and were left without additional treatment till week 8 when they were immunized. Control (Ctrl) rats were maintained in parallel. The treated and Ctrl rats were offspring of littermate male and littermate female rats.

### Gut Microbiota Transfer

For gut microbiota transfer (fecal transplantation), cecal content obtained from adult healthy untreated AO rats (2–4 months old) was dissolved in physiological saline (one cecum per 40 ml) and filtered through filter paper. This was further diluted with drinking water and applied to DA rats *ad libitum*. Ctrl DA rats had the same amount of physiological saline in their drinking water. Also, feces obtained from AO rat beddings was transferred into clean DA rat beddings. The combined treatment started 1 week before birth of pups and lasted for 12 weeks. Ctrl rats did not receive feces from AO rat beddings. The rats were immunized at age of 8 weeks. The treated and Ctrl rats were offspring of littermate male and littermate female rats.

### Denaturing Gradient Gel Electrophoresis (DGGE) Analysis and DNA Sequencing

Feces was collected from rats on separation (4 weeks of age), on the day of immunization (8 weeks of age) and after the immunization (9 and 10 weeks of age) and stored at −20°C. Extraction of bacterial DNA from frozen fecal samples was done using the QIAamp DNA stool minikit (Qiagen, Hilden, Germany). DGGE analysis and gel manipulation after electrophoresis was entirely performed as described previously (17). The primer set Lab-0159f and Uni-0515-GCr (Metabion International, Martinsried, Germany), complementary to lactobacilli and related lactic acid bacteria (LAB), as well as the universal primer set U-968-GC-f pared with L1401-r, complementary to 16S rDNA specific for Eubacteria were used (18, 19). Fragments of interest were excised from the gel and macerated, and the suspension was incubated for 10 min at 98°C (17). After incubation, the suspension was centrifuged to pellet gel particles. The supernatant (30 µl) was used in polymerase chain reaction (PCR) with Lab-0159f and Uni-0515GCr primers (18). The obtained PCR products were purified using the QIAquick PCR purification kit (Qiagen) and ligated into the pJET1.2/blunt vector (CloneJET PCR Cloning Kit, Thermo Scientific). Ligated constructs were transformed in Ca_2_-induced competent DH5α cells (20), and insert-containing transformants were selected as white colonies on Luria-Bertani agar plates containing 100 µg/ml ampicillin and 20 µg/ml X-Gal (5-bromo-4-chloro-3-indolyl-β-d-galactoside) as recommended by Promega. For each excised DNA band, one white colony was picked and plasmids were isolated using the QIAprep spin miniprep kit (Qiagen). The sequencing of the isolated insert-containing pJET1.2/blunt plasmids was done with pJET1.2 sequencing primers at Macrogen Europe Service, Amsterdam, Netherlands. Sequence annotation and the database searches for sequence similarities were performed with the BLAST tool available online.

### Isolation of Cells, Cell Culturing, and Generation of Supernatants

Cells of lymph nodes draining the site of immunization (PLN) were obtained from rats immunized with 100 µl of emulsion made of MBP and CFA, as described above. PLN cells (PLNC) were obtained by mechanical disruption. The isolation took place at 4, 7, and 14 days post immunization (d.p.i.). Mesenteric lymph nodes (MLNs) and Peyer’s patches (PP) were isolated from non-immunized rats and from rats on 4, 7, and 14 d.p.i. Four MLN were isolated from each rat and MLN cells (MLNC) were prepared by mechanical disruption. PP were obtained from the small intestine and PP cells (PPC) were obtained by mechanical disruption. PLNC were cultured in RPMI 1640 culture medium (PAA Laboratories, Pasching, Austria) that was supplemented with 2% rat serum. The cells were seeded at 5 × 10^6^/ml/well in 24-well plates (Sarstedt, Nümbrecht, Germany) and stimulated with MBP (10 µg/ml). MLNC and PPC were grown in RPMI1640 medium supplemented with 5% fetal calf serum (PAA Laboratories). MLNC (2.5 × 10^6^/ml) and PPC (2 × 10^6^/ml) were stimulated with concanavalin A (ConA, Sigma-Aldrich, 2.5 µg/ml). Cultures lasted for 24 h and subsequently cell culture supernatants were collected and kept frozen until assayed. Supernatants were also obtained from SCHs (1 g of spinal cord homogenized in 2 ml of PBS) after centrifugation on 10,000 *g* for 20 min.

### Cytofluorimetry

Cells were stained with the following antibodies: PE-conjugated anti-CD4 (mouse monoclonal OX35, eBioscience) and FITC-conjugated anti-CD25 (mouse monoclonal NDS601, AbD Serotec, Oxford, UK). PE/Cy5-conjugated FoxP3 staining with rat monoclonal antibody (FJK-16s) was performed according to the procedure suggested by the manufacturer (eBioscience). Appropriate isotype Ctrl antibodies were used where necessary to set gates for cell marker positivity. Typically, proportion of isotype Ctrl antibody-stained cells was <1%. Cells were gated to live cells (gate R1 based on FSC/SSC). Proportion of FoxP3^+^ cells was determined among CD4^+^CD25^+^ cells (gate Q2). Analyses were performed on a Partec CyFlow Space cytometer (Partec, Munster, Germany). Results of cytofluorimetry are presented as proportion of cells bound by an appropriate antibody.

### ELISA

Cytokine concentration in supernatants was determined by sandwich ELISA using MaxiSorp plates (Nunc, Rochild, Denmark). For IL-10 detection Rat IL-10 DuoSet ELISA was used according to the manufacturer’s instructions (R&D Systems, Minneapolis, MN, USA). For IFN-γ and IL-17 detection anti-cytokine paired antibodies were used according to the manufacturer’s instructions (eBioscience, San Diego, CA, USA). The antibodies were as follows: anti-rat IFN-γ purified mouse monoclonal (DB1), anti-rat IFN-γ biotinylated rabbit polyclonal, anti-mouse/rat IL-17A purified rat monoclonal (eBio17CK15A5), and anti-mouse/rat IL-17A biotinylated rat monoclonal (eBio17B7). Samples were analyzed in duplicates and the results were calculated using standard curves made on the basis of known concentrations of the recombinant rat IL-10 (R&D Systems) and IFN-γ and IL-17 (Peprotech, Rocky Hill, NJ, USA).

### Statistical Analysis

Two-way ANOVA followed with Student’s *t*-test (two-tailed) or Mann–Whitney *U* test, as appropriate was performed for statistical analysis. A *p*-value less than 0.05 was considered statistically significant. Differences in DGGE profiles of gut microbiota in various animal groups were calculated by Dice similarity coefficient. Similarity between single groups was determined as:

Dsc=2j×100/a+b,

where, *a* is the number of DGGE bands in one group; *b* is the number of DGGE bands in second group; *j* is the number of the same bands in both groups. Dsc = 100 represents completely identical DGGE profiles, while Dsc = 0 represents completely different DGGE profiles.

## Results

### Antb Treatment Changes Gut Microbiota Composition in AO Rats

**Figure 1:**
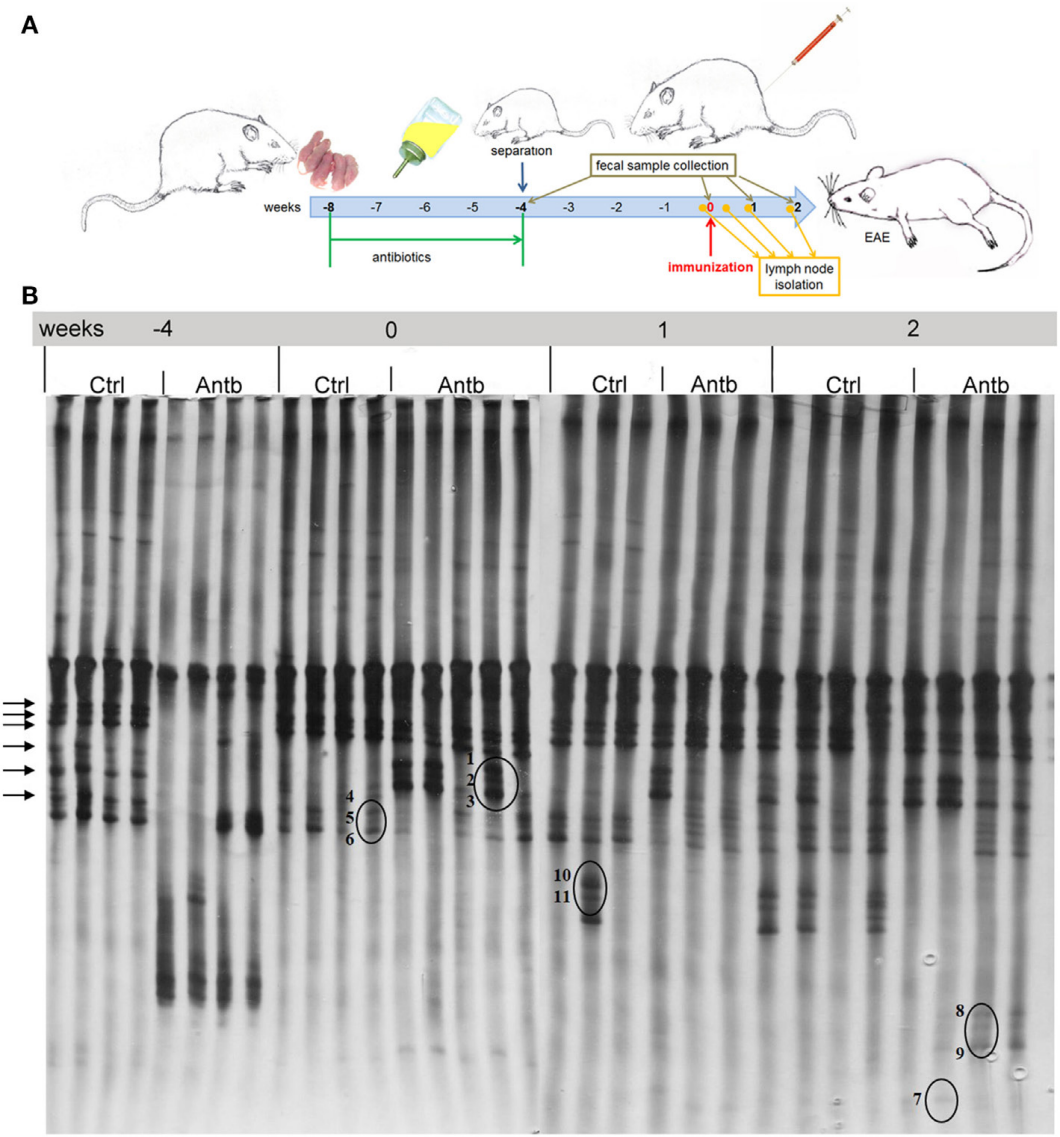
Effects of antibiotic (Antb) treatment on Albino Oxford (AO) rat gut microbiota composition. AO rats, untreated [control (Ctrl)] or treated with Antb, were immunized with spinal cord homogenate + complete Freund’s adjuvant and samples were collected as depicted **(A)**. Denaturing gradient gel electrophoresis (DGGE) profiles of rDNA amplicons obtained using a *Lactobacillus-*specific primer set on bacterial DNA isolated from fecal samples of AO rats **(B)**. Each lane represents sample of an individual rat. Arrows represent DGGE bands present in Ctrl samples and absent in samples from Antb-treated rats at 4 weeks of age. The annotated species of the sequenced DGGE bands (1–11) are given in Table 1.

Albino Oxford rats were treated with Antb to perturb their gut microbiota. The treatment started at the day of birth and lasted for 4 weeks. Thus, pups were exposed to Antb indirectly through dams. Fecal samples were collected at the point of weaning which corresponded to cessation of Antb treatment (4 weeks of age), as well as at the time of immunization (0 d.p.i., 8 weeks of age). Fecal samples were also collected after SCH + CFA immunization in the inductive phase of EAE (7 days p.i., 9 weeks of age) and at the time of clinically manifested disease (14 days p.i. 10 weeks of age). DGGE analysis of rDNA amplicons using DNA isolated from fecal samples as templates and Lab-0159f and Uni-0515GCr primer set was performed. The treatment led to the change in DGGE profile of gut microbiota at week 4, where specific DGGE bands representing core microbiota in Ctrl samples (e.g., *Lactobacillus murinus* and *Lactobacillus intestinalis*, Table 1) were absent in samples from Antb-treated rats (Figure 1). However, the changes were less prominent at weeks 8, 9, and 10, due to relative heterogeneity of the samples (Figure 1). Thus, Antb treatment led to transient change in gut microbiota composition in AO rats. Specific differences that were determined by sequencing are presented at Figure 1, while annotated bacterial species are given in Table 1. Presence of *L. murinus* in Antb-treated rats and of *L. intestinalis* in Ctrl rats at 0 d.p.i. suggests that *L. murinus* colonizes in opportunistic way, when competition is reduced. Still the differences in the presence of *L. intestinalis* are lost at 14 d.p.i. Interestingly, *Roseburia* sp., butyrate producing commensal bacteria and *Lactobacillus gasseri* were observed exclusively in Antb-treated rats at 7 and 14 d.p.i.

**Table 1:** Bacteria sequences.

No. of band	Species determined by sequencing of 16S rDNA	Identity (%)
1	*Lactobacillus murinus* strain NBRC 14221 16S ribosomal RNA gene, partial sequence	99
2	*L. murinus* strain NBRC 14221 16S ribosomal RNA gene, partial sequence	100
3	*L. murinus* strain NBRC 14221 16S ribosomal RNA gene, partial sequence	99
4	*Lactobacillus intestinalis* strain TH4 16S ribosomal RNA gene, partial sequence	98
5	*L. intestinalis* strain TH4 16S ribosomal RNA gene, partial sequence	99
6	*L. intestinalis* strain TH4 16S ribosomal RNA gene, partial sequence	99
7	*Lactobacillus gasseri* strain ATCC 33323 16S ribosomal RNA gene, complete sequence	99
8	*L. gasseri* strain ATCC 33323 16S ribosomal RNA gene, complete sequence	99
9	*Roseburia intestinalis* strain DSM 14610 16S ribosomal RNA gene, partial sequence*/Roseburia inulinivorans* strain A2-194 16S ribosomal RNA gene, complete sequence*/Roseburia hominis* strain A2-183 16S ribosomal RNA gene, complete sequence/*Roseburia faecis* strain M72/1 16S ribosomal RNA gene, partial sequence (84%) *Roseburia* sp.	84
10	*L. intestinalis* strain TH4 16S ribosomal RNA gene, partial sequence	99
11	*Lactobacillus faecis* strain AFL13-2 16S ribosomal RNA gene, partial sequence	99

### Antb Treatment Affects PLNC

**Figure 2:**
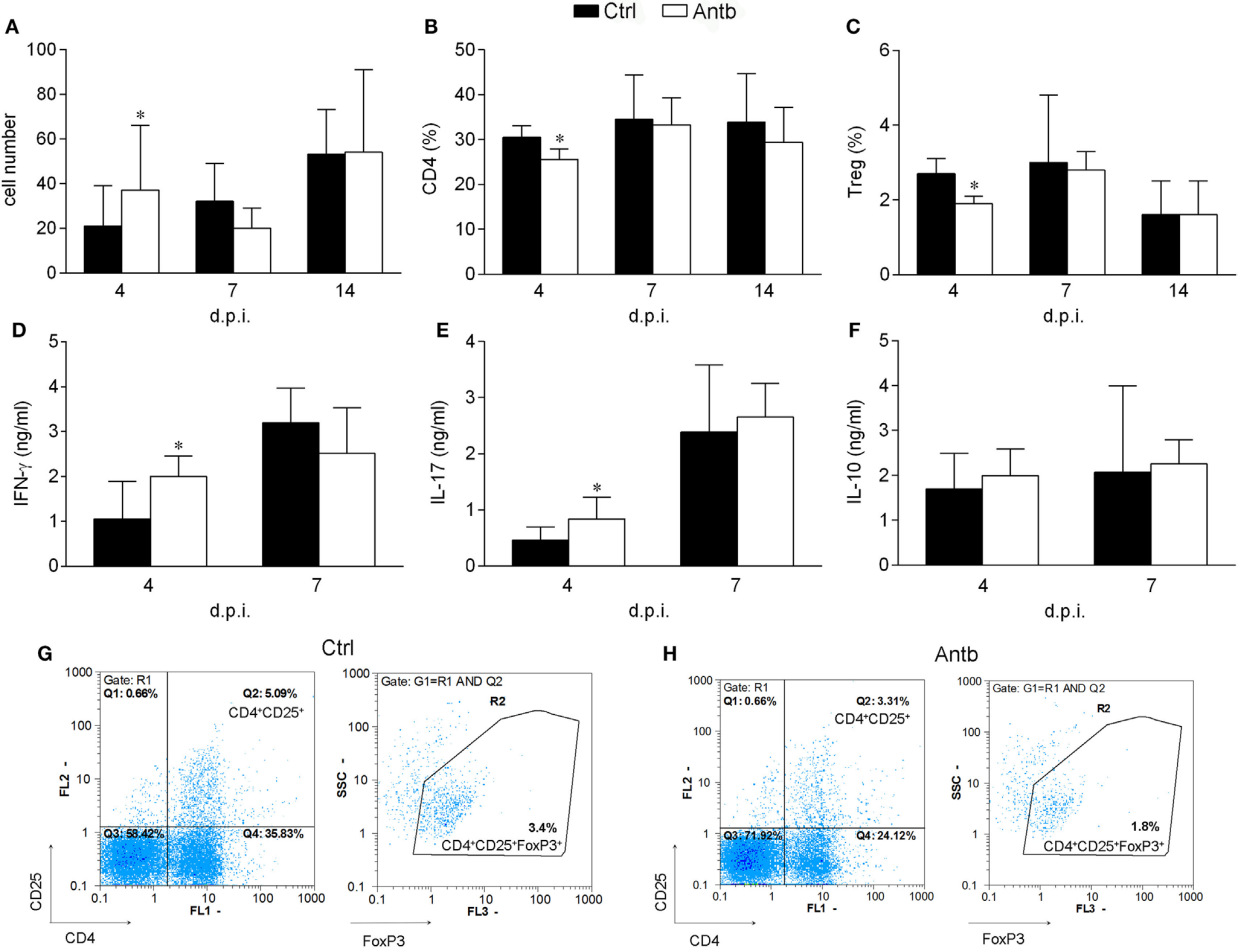
Effects of antibiotic (Antb) treatment on PLN cells (PLNC). Albino Oxford rats were untreated [control (Ctrl)] or treated with Antb and they were immunized with spinal cord homogenate + complete Freund’s adjuvant (CFA) **(A–C,G,H)** or with myelin basic protein + CFA **(D–F)**. PLNC were isolated from rats at 4, 7, and 14 days post immunization (d.p.i.). Cellularity **(A)**, CD4 proportion **(B)**, regulatory T cell (Treg) proportion **(C)**, interferon (IFN)-γ levels **(D)**, IL-17 levels **(E)**, and IL-10 levels **(F)** were determined. The total number of samples obtained in at least three independent experiments was 20 **(A)**, 24 **(B,C)**, and 9 **(D–F)** per group. Data are presented as mean ± SD. Representative plots obtained at 4 d.p.i. are showing gating strategy for Treg **(G,H)**. *p < 0.05 Antb vs. Ctrl (*t*-test).

Cellularity, phenotypic analysis, and MBP-stimulated cytokine production of PLN was compared in Antb-treated and Ctrl AO rats at different time points after the immunization. Number of cells per PLN was significantly higher in Antb-treated rats at 4 d.p.i., but not at 7 and 14 d.p.i. (Figure 2A). Percentage of CD4^+^CD25^+^Foxp3^+^ regulatory T cells (Treg) was reduced in Antb-treated rats at 4 d.p.i. (Figures 2B,C,G,H). At the same time, higher levels of IFN-γ and IL-17, but not of IL-10 were observed in MBP-stimulated PLNC obtained from Antb-treated rats (Figures 2D–F).

### Antb Treatment Affects MLNC and PPC

**Figure 3:**
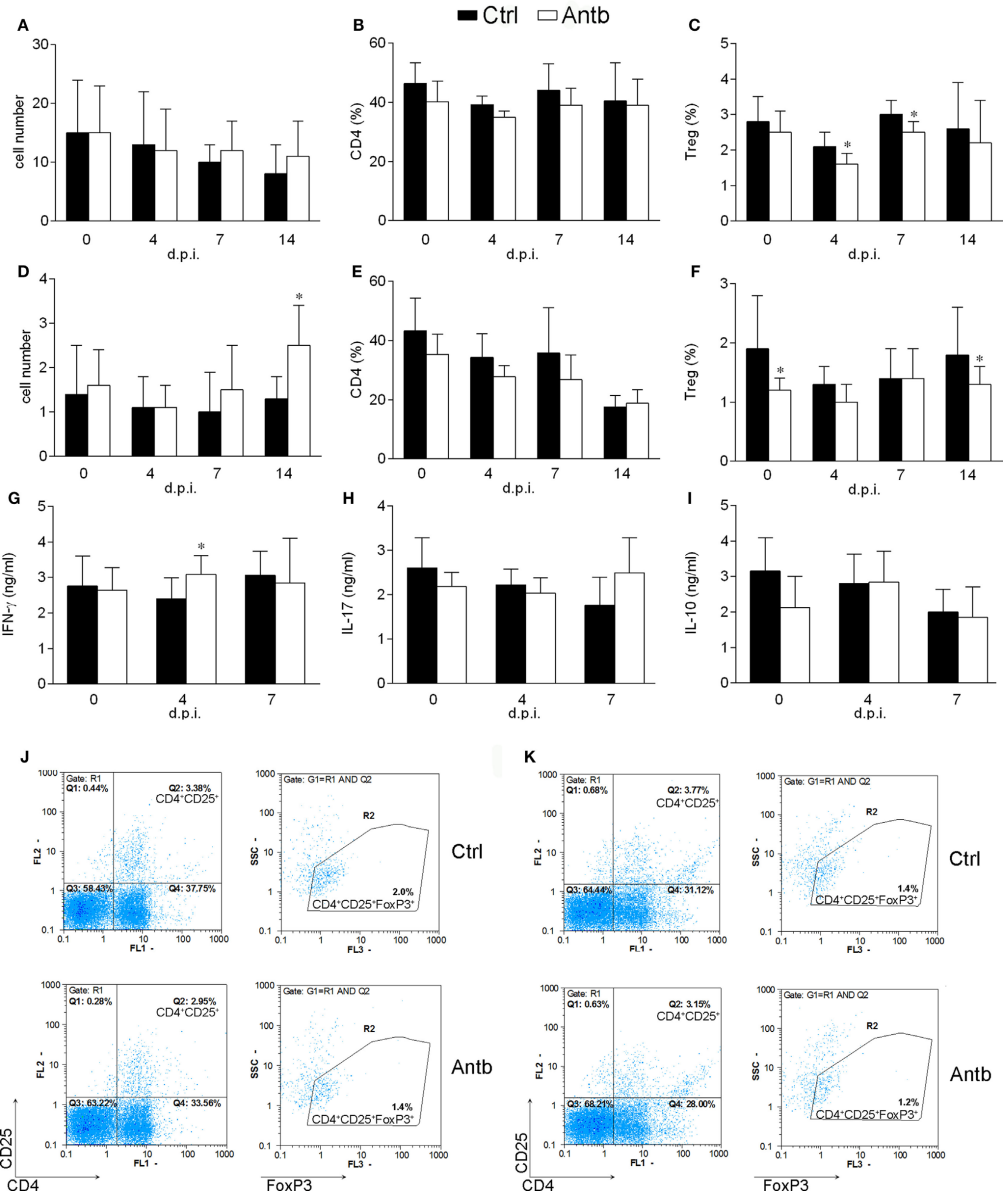
Effects of antibiotic (Antb) treatment on MLN cells (MLNC) and PP cells (PPC). Albino Oxford rats were untreated [control (Ctrl)] or treated with Antb. MLNC **(A–C,G–I,J)** and PPC **(D–F,K)** were isolated from rats at 0, 4, 7, and 14 days post immunization (d.p.i.) (spinal cord homogenate + complete Freund’s adjuvant). Cellularity **(A,D)**, phenotype **(B,C,E,F)**, and cytokine generation **(G–I)** were determined. The total number of samples obtained in at least three independent experiments was 12 **(A,C)**, 24 **(B,C,D,E)**, 7 **(G–I)** per group. Data are presented as mean ± SD. Representative plots obtained at 4 d.p.i. are showing gating strategy for regulatory T cells (Treg) in MLNC **(J)** and PPC **(K)**. *p < 0.05 Antb vs. Ctrl (*t*-test).

Cellularity, phenotypic analysis, and ConA-stimulated cytokine production of MLN and PP were determined in Antb-treated and Ctrl non-immunized AO rats, as well as at different time points after the immunization. Number of cells and proportion of CD4^+^ cells in MLN and PP was without difference in Ctrl and Antb-treated rats in all examined time points (Figures 3A,B,D,E). Percentage of Treg was lower in MLN of Antb-treated rats on 4 and 7 d.p.i., and in PP before the immunization, at day 4 d.p.i. and at 14 d.p.i. (Figures 3C,F,J,K). Production of IFN-γ, IL-17, and IL-10 was without difference in MLN of Ctrl and Antb-treated rats before the immunization and on 7 d.p.i., yet production of IFN-γ, but not the other two cytokines was significantly higher in Antb-treated rats on 4 d.p.i. (Figures 3G–I).

### Antb Treatment Induces EAE in AO Rats

**Figure 4:**
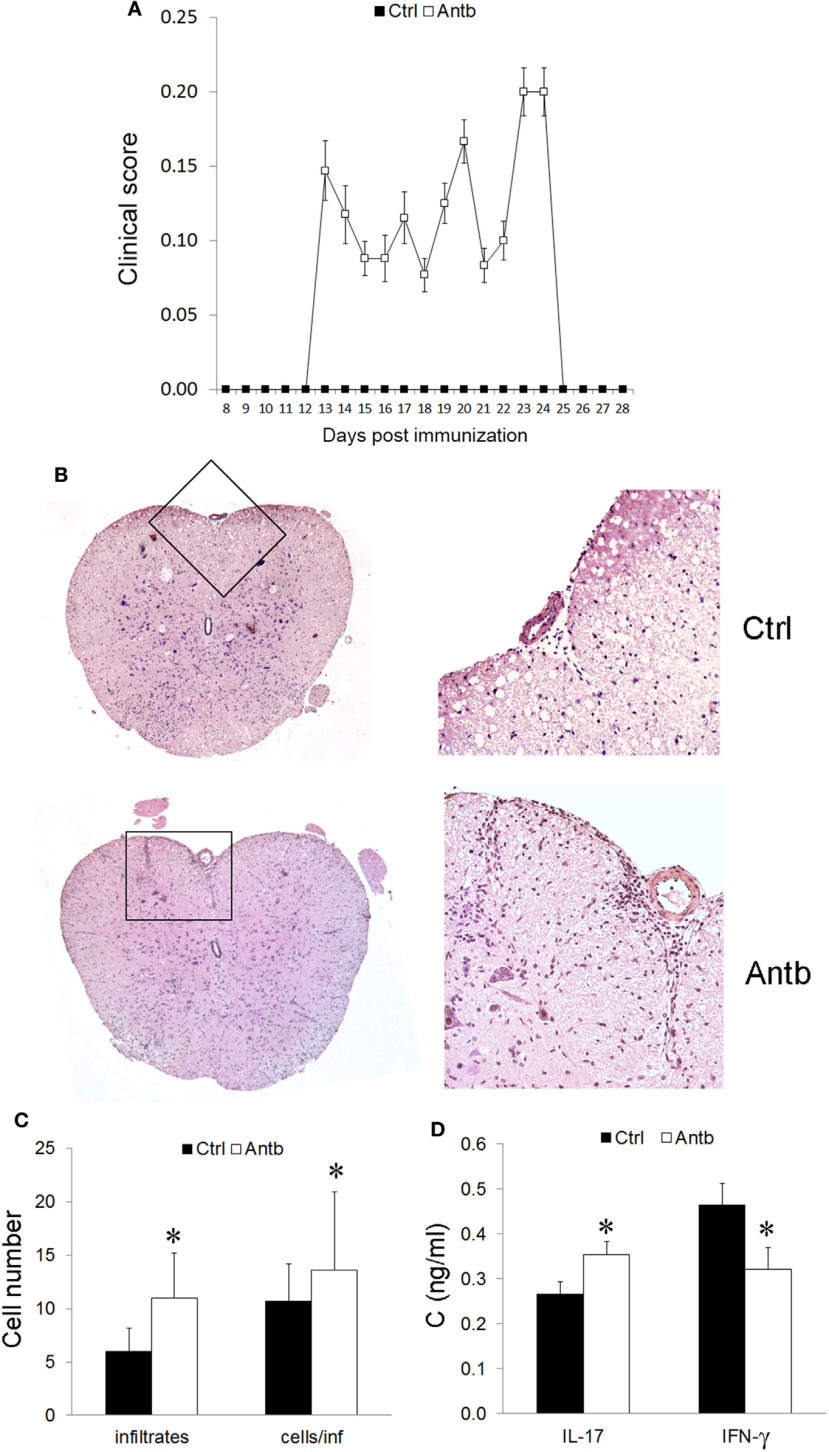
Effects of antibiotic (Antb) treatment on experimental autoimmune encephalomyelitis. Albino Oxford rats were untreated [control (Ctrl)] or treated with Antb. They were immunized with spinal cord homogenate + complete Freund’s adjuvant and clinical score was determined daily **(A)**. Results are presented as mean ± SE of 20 Ctrl and 17 Antb rats in total, from two independent experiments. Spinal cords were isolated at 15 days post immunization and stained with hematoxylin. Representative spinal cord sections are presented **(B)**. Number of infiltrates and number of cells per infiltrate is presented as mean ± SD from the total number of 120 sections obtained from five rats per group **(C)**. Level of cytokines was determined in spinal cord homogenates. The total number of samples obtained in two independent experiments was nine per group **(D)**. Results are presented as mean ± SD. *p < 0.05 Antb vs. Ctrl (*t*-test).

In order to test if the observed changes in gut microbiota, PLN, MLN, and PP have any relevance for pathogenesis of EAE the groups of Antb-treated and untreated rats, both immunized with SCH-CFA were followed for 40 days in order to evaluate clinical and histological manifestations of disease. Unlike Ctrl untreated rats that were uniformly without any clinical signs during the observation period, more than 50% of Antb-treated rats expressed mild clinical signs of EAE (Table 2; Figure 4A). The broken resistance to EAE was paralleled with more numerous spinal cord infiltrates and higher number of cells per infiltrate (Figures 4B,C). Also, level of IL-17 was significantly higher in supernatants of SCHs obtained from Antb-treated AO rats (Figure 4D). Interestingly, IFN-γ level was significantly reduced in these supernatants (Figure 4D).

**Table 2:** Effects of antibiotic (Antb) treatment on Experimental Autoimmune Encephalomyelitis.

	Incidence	Duration (days)	Mean c.s.	Cumulative c.s.	Maximal c.s.
Control	0/20	0	0	0	0
Antb	9/17	3.0 ± 1.9	0.6 ± 0.1	1.8 ± 1.3	0.7 ± 0.3

*Results are presented as mean ± SD*.

*c.s., clinical score*.

### AO Rat Gut Microbiota Transfer Ameliorates EAE in DA Rats

**Figure 5:**
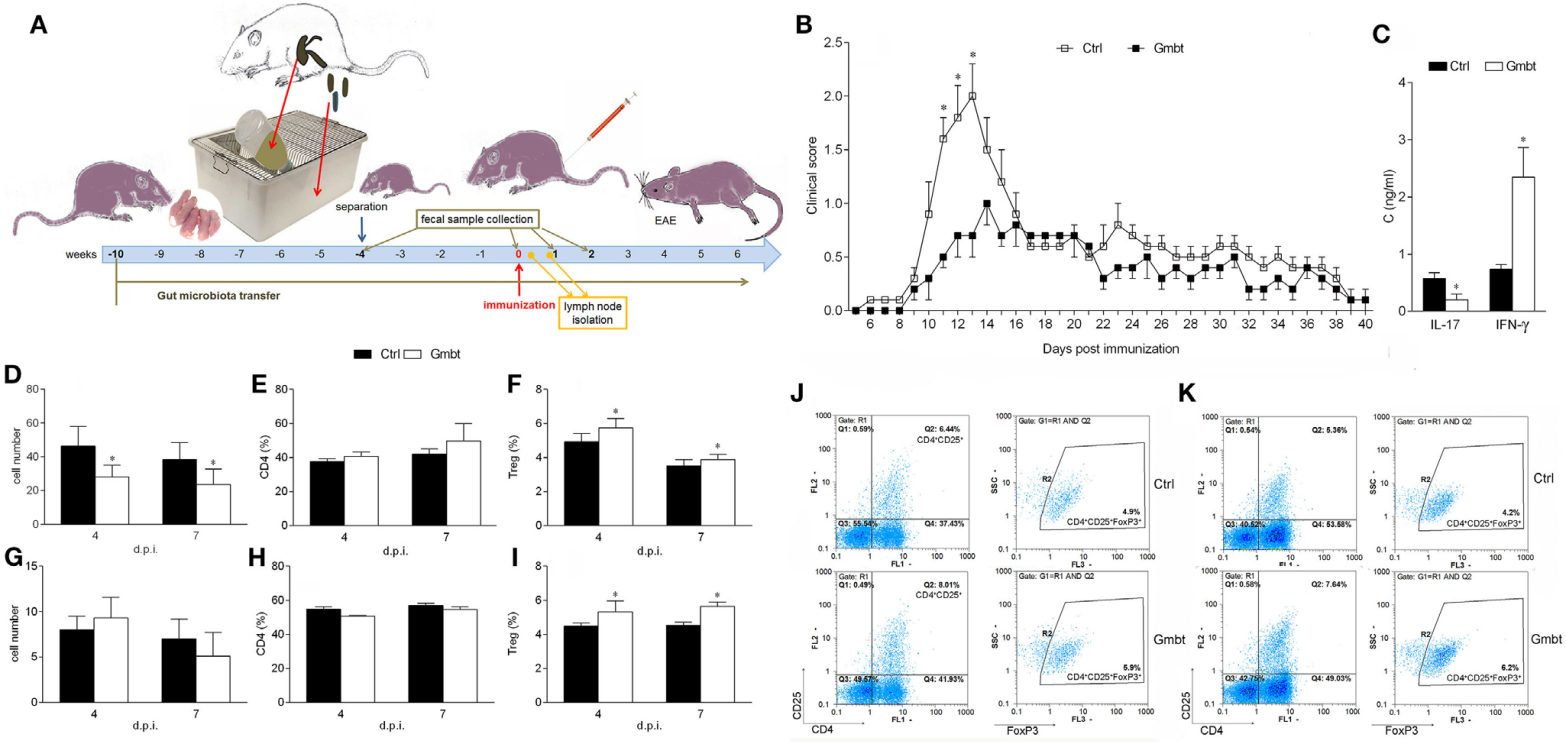
Effects of Albino Oxford (AO) gut microbiota transfer on experimental autoimmune encephalomyelitis in Dark Agouti (DA) rats. DA rats were untreated [control (Ctrl)] or treated with AO gut microbiota (Gmbt) as represented in scheme **(A)**. They were immunized with spinal cord homogenate + complete Freund’s adjuvant and clinical score was determined daily **(B)**. The total number of samples obtained in two independent experiments was 14 per group. Results are presented as mean ± SE. Spinal cords were isolated at 14 days post immunization (d.p.i.) and cytokines were measured in supernatants obtained by centrifugation of spinal cord homogenates **(C)**. The total number of samples obtained in two independent experiments was four per group. Results are presented as mean ± SD. Popliteal lymph node (PLN) **(D–F)** and mesenteric lymph node **(G–I)** were isolated at 4 and 7 d.p.i. Cellularity **(D,G)**, percentage of CD4^+^ T cells **(E,H)** and regulatory T cells (Treg) **(F,I)** were determined. The total number of samples obtained in two independent experiments was five **(D,E,G,H)**, and nine **(F,I)** per group. Results are presented as mean ± SD. Representative plots obtained at 4 d.p.i. are showing gating strategy for Treg in PLN cells (PLNC) **(J)** and MLN cells (MLNC) **(K)**. *p < 0.05 Gmbt vs. Ctrl [*U* test **(B)**, *t*-test **(C–I)**].

Finally, to investigate potential beneficial effects of AO gut microbiota, its transfer into EAE-prone DA rats was performed. DA rats were continuously exposed to AO gut microbiota from birth. EAE was induced when rats were 8 weeks of age (Figure 5A). The disease was ameliorated in AO gut microbiota-transferred DA rats in comparison to untreated counterparts (Figure 5B; Table 3). IL-17, but not IFN-γ level was decreased in SCHs of the treated rats (Figure 5C). The treatment led to decrease in number of PLNC in the inductive phase of the disease (4 and 7 d.p.i.) (Figure 5D). There was no difference in cellularity of MLN (Figure 5G), nor in proportion of CD4^+^ T cells in lymph nodes (Figures 5E,H). Importantly, proportion of Treg was increased both in draining and MLNs at the same time (Figures 5F,I,J,K).

**Table 3:** Effects of Albino Oxford gut microbiota transfer on experimental autoimmune encephalomyelitis in Dark Agouti rats.

	Incidence	Onset	Duration (days)	Mean c.s.	Cumulative c.s.	Maximal c.s.
Ctrl	15/15	9.9 ± 1.1	23.6 ± 6.5	0.9 ± 0.2	22.6 ± 9.6	2.4 ± 1.0
Gmbt	13/13	12.2 ± 5.3	18.6 ± 7.6	0.7 ± 0.2*	14.2 ± 8.2*	1.6 ± 0.7*

*Results are presented as mean ± SD. c.s., clinical score*.

*p < 0.05 Gmbt vs. Ctrl (t-test).

**Figure 6:**
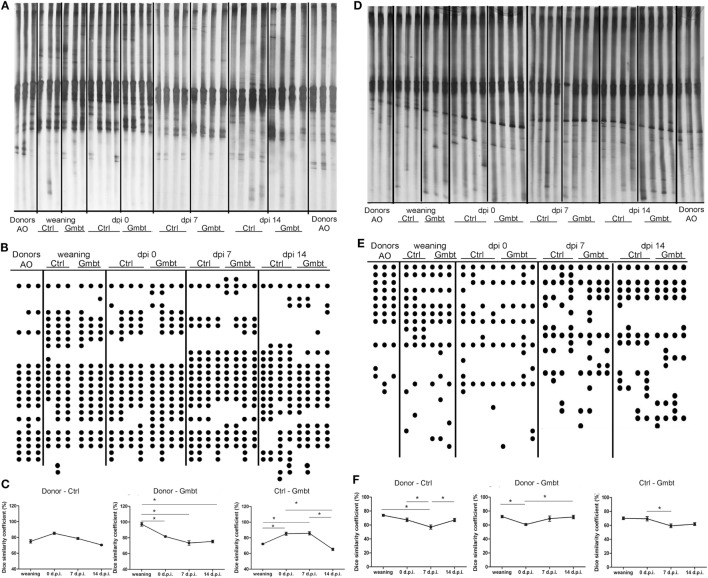
Effect of Albino Oxford (AO) gut microbiota transfer to Dark Agouti (DA) rats on microbiota diversity. DA rats were untreated [control (Ctrl)] or treated with AO gut microbiota (Gmbt) as represented in Figure 5A. They were immunized with spinal cord homogenate + complete Freund’s adjuvant and fecal samples were collected at the time of weaning, at the time of immunization [0 days post immunization (d.p.i.)], as well as at two time points after the immunization (7 and 14 d.p.i.). Denaturing gradient gel electrophoresis (DGGE) **(A,D)**. The clearly visible bands were represented as DGGE profiles scheme of rDNA amplicons **(B,E)** where each dot represents a corresponding band on DGGE gel. DGGE gel and profiles obtained using *Lactobacillus* sp.-specific primers **(A,B)** and DGGE gel and profiles obtained using universal primers **(D,E)** on bacterial DNA isolated from fecal samples of donor AO rats (Donor), AO gut microbiota-transferred DA rats (Gmbt) and Ctrl animals (Ctrl). Each lane represents sample of an individual rat. Box-plot diagram based on Dice similarity coefficient (*p < 0.05) **(C,F)**.

In order to determine the influence of AO gut microbiota transfer on DA gut microbiota, DGGE analysis of rDNA amplicons obtained by using DNA isolated from fecal samples of AO (Donor) and DA (gut microbiota-transferred—Gmbt and Ctrl) rats as templates and *Lactobacillus* sp.-specific primers complementary to 16S of lactobacilli and related LAB (Figures 6A–C) and universal primers complementary to 16S rDNA regions of Eubacteria (Figures 6D–F). The presence of specific clearly visible bands in DGGE profiles was analyzed by Dice similarity coefficient. The results of the analysis revealed that the transfer of AO gut microbiota to DA rats significantly (*p* < 0.05) influenced the diversity of gut microbiota in the treated DA rats (Figures 6C,F). Particularly, LAB diversity of the DA rats after gut microbiota transfer (Gmbt) was significantly (*p* < 0.05) different comparing to Ctrl animals in the period from weaning to the day of immunization. At the same time, Dice coefficient was significantly higher for AO and Gmbt group (nearly 100%), which points to the successful transplantation of LAB strains from AO to Gmbt group. Four weeks after the weaning the similarity in LAB strains composition between AO and Gmbt groups starts to decrease, but at the same time the coefficient starts to increase between Ctrl and Gmbt groups pointing to the returning of LAB composition to the composition in the Ctrl group. Additionally, the similarity of LAB composition between these two groups starts to decrease again at 14 d.p.i., which is probably the effect of immune mechanisms on microbiota composition (Figure 6C). Similarly, the total gut microbiota diversity of Gmbt DA rats was significantly (*p* < 0.05) different comparing to Ctrl animals (Ctrl) in the period from 0 to 7 d.p.i., while the similarity between AO and Ctrl DA rats is the lowest at 7 d.p.i. indicating that this difference could be crucial for the disease prognosis (Figure 6F).

## Discussion

The results of this study show that prolonged oral application of Antb to AO rats modifies their gut microbiota and GALT. Subsequently, this treatment stimulates encephalitogenic immune response and overcomes resistance of AO rats to EAE. Although we do not exclude other possible reasons (genetic, hormonal) for the extreme resistance of AO rats to EAE induction observed in our previous studies (14, 21), the observed annulation of the resistance through the Antb treatment is a compelling result despite mild clinical symptoms that were achieved. Gut microbiota-independent effects of Antb on EAE susceptibility in AO rats cannot be disregarded. However, the substantial modification of AO gut microbiota determined after the Antb treatment allows us to assume that the observed effects of Antb on EAE susceptibility in AO rats are, at least partly, gut microbiota-mediated. These effects were associated with reduction of regulatory T cells (Treg) and upregulation of pro-inflammatory components in lymph organs and within the CNS. Importantly, the effects were mostly observed in early inductive phase of the disease (4 d.p.i.), i.e., at the time when the autoimmune response was generated. This implies that gut microbiota is an important factor of AO rat resistance to EAE induction. However, no such distinctive differences in gut microbiota have been observed at the time when the effect on Treg was detected, indicating that the effect on immune system at 4 d.p.i. could be a consequence of Antb-induced gut microbiota dysbiosis in early-life period (4 weeks of age). It is becoming increasingly appreciated that early microbial gut colonization highly influences life-long health through the interaction with the immune system, while the failure in proper immune modulation and induction of immunological tolerance could result in development of autoimmune diseases (22).

Gut microbiota and GALT has been proposed as important players in initiation, propagation, but also in prevention of CNS autoimmunity (4). Indeed, gut microbiota composition is different in multiple sclerosis patients and healthy individuals (7–10). Accordingly, AO rats that are EAE-resistant have different gut microbiota composition in comparison to EAE-prone DA rats (15, 16). Also, our previous study showed that cellular composition of MLN and PP differ between AO and DA rats, the latter having more CD4^+^ cells and even more Treg (15). Here, we present evidence that disturbance of gut microbiota by Antb makes AO rats susceptible to EAE induction. There were numerous reports on the effect of Antb treatment on EAE that did not focus on gut microbiota, but instead used Antb to affect other EAE pathogenesis-related components (23–28). Still, there were also reports focused on gut microbiota that gave significant contribution to our understanding of the role of intestinal microbiota in EAE (29, 30). They all presented beneficial effects of Antb treatment in EAE. Our results show that Antb can also be detrimental in the CNS autoimmunity as AO rats that are resistant to EAE develop clinically manifested disease upon Antb treatment. These contradictory results could be explained in several ways. For instance, the data showing disease-attenuating effects of Antb are based on the treatment of adult animals while disease-promoting effect demonstrated in our study resulted from the Antb application that started in the neonatal period. It is well established that environmental factors, nutrients, infections, and Antb in early-life lead to microbiota alteration with considerable consequences on the development and function of immune system [reviewed in Ref. (31)]. Indeed, similar results were obtained in experimental psoriasis in mice where neonatal treatment with Antb increased susceptibility to the disease, while the same treatment of adult animals ameliorated this disease (32). Also, numerous studies have revealed that different Antb induce distinct changes in gut microbiota [reviewed in Ref. (33)], which consequently may result in different modulation of the immune responses. It has recently been shown that neomycin treatment resulted in protection from autoimmune diabetes while vancomycin induced more diabetogenic response in NOD mice (34). Thus, the difference in the outcome of Antb treatment, i.e., EAE amelioration described by others vs. breakdown of EAE resistance which we demonstrate, might be partly ascribed to the use of different combination of Antb. Indeed, it has recently been suggested by Qinghui et al. (35), working on gut microbiota modulation in a systemic lupus erythematosus animal model, that the appropriate choice of Antb that selectively target harmful bacteria is of crucial importance for amelioration of the disease. Importantly, the disease was not altered in germ-free mice, thus showing that complete absence of gut microbiota did not have any effect on the lupus-like disease in mice. In our study, the prolonged Antb treatment caused considerable gut microbiota perturbation, apparently with absence of major bacterial groups, similar to the germ-free status. Particularly, members of the genus *Roseburia* were detected in Antb-treated rats at 14 d.p.i. The perturbations of *Roseburia* spp., previously correlated to immunity maintenance and anti-inflammatory properties in gut, was recently correlated to various diseases, including irritable bowel syndrome, obesity, Type 2 diabetes, nervous system conditions and allergies, while the presence of *Roseburia* spp. showed beneficial effect as probiotic for restoration of beneficial flora (36). Interestingly, the gut microbiota diversity was mainly restored during post-Antb period, although some specific bacterial groups were altered, in comparison to Ctrl groups. Taking into account the appearance of the disease symptoms in otherwise EAE-resistant AO rats, it could be concluded that the altered microbiota containing higher abundance of harmful bacteria correlated with the promotion of autoimmunity and the diseases symptoms.

Autoimmunity-promoting effects of Antb have been demonstrated before. Besides above mentioned experimental psoriasis (32), Antb exacerbated neurological signs and lesions of infection induced Guillain–Barré Syndrome in NOD mice (37). Also, Antb treatment favored priming of diabetogenic T cells, thus promoting disease progression in NOD mice (38) and reestablished expected incidence of diabetes in mice previously infected with segmented filamentous bacteria to prevent the development of autoimmunity (39). Although simultaneous application of different Antb in high doses and for extremely long period is not likely to be used in humans, our results call upon caution in Antb use. Importantly, our results were obtained from rats treated with Antb from their birth till week 4 of their age, i.e., well before the immunization (week 8 of their age). This suggests that gut microbiota disturbance in the early childhood can predispose an individual toward CNS autoimmunity that can be provoked by independent stimuli at the latter age. Indeed, differences in rat gut microbiota were prominent at the end of the Antb treatment (4 weeks of age), but they were almost absent at the time of immunization (8 weeks of age). This implied that EAE-promoting effects of Antb were achieved through gut microbiota perturbance in the early-life, possibly through effect on adaptive immune system development. Accordingly, there were less Treg in PP of these rats. Therefore, deeper investigation of the effects of early postnatal application of Antb on the GALT is needed. Also, our data call upon epidemiological studies that will explore gut microbiota perturbances as a consequence of Antb treatment in the early childhood of multiple sclerosis patients. Indeed, Antb-induced dysbiosis in children has been already proposed as a potential predisposing factor for various diseases, including autoimmune ones (40).

It is plausible to assume that the mechanism behind EAE-promoting effects of Antb observed in AO rats is the functional consequence of changes in microbial composition that deregulated Th1- and Th17-driven immune response. Indeed, draining lymph node cells of Antb-treated rats produced more IFN-γ and IL-17, marker cytokines of Th1 and Th17, respectively. This probably came as a consequence of reduced Treg response as lower Treg proportion was observed in draining lymph nodes, MLNs, and PP after the immunization. This hypothesis is in accordance with previous reports on gut microbiota-related effects on EAE. For instance, group of Kasper showed in a series of papers that beneficial effects of gut microbes on EAE were achieved through generation of Tregs that prevented encephalitogenic activity of Th cells (41–44). Along the same line, increase in Treg potency and subsequent downregulation of Th1/Th17 encephalitogenic activity is likely mechanism for the observed beneficial effects of AO gut microbiota transfer in DA rats EAE. As a consequence of the transfer, increased proportion of Treg was observed in draining and MLNs of DA rats after the immunization.

Noteworthy, not only that gut microbiota contributed to the resistance of AO rats to the CNS autoimmunity, but its transfer into DA rats ameliorated EAE in the recipients. Although the similarity in LAB composition between AO and Gmbt DA rats was the lowest at 7 d.p.i., when the disease amelioration was observed in Gmbt DA rats compared to Ctrl animals, it could be referred as the positive impact of AO gut microbiota transfer to DA rats during the first days of life. The success of the gut microbiota transfer from EAE-resistant rats to EAE-prone rats implies that similar therapeutic approach might be useful in multiple sclerosis. Importantly, future studies should determine if EAE-restricting effects of gut microbiota are specific for AO rats or similar effects could be obtained by transfer of gut microbiota from other rat strains, or even with autologous gut microbiota transfer. Also, the upcoming studies should investigate if AO gut microbiota has beneficial effects in other autoimmune and chronic inflammatory disorders. Gut microbiota transfer seems like a promising strategy for the treatment of gut microbiota-related disorders, including multiple sclerosis (45). Still, in our experiments DA rats were exposed to AO rat gut microbiota from their birth to the time of EAE immunization. Such a gut microbiota transfer will be feasible in humans only as preventive therapy when genetic prediction markers for multiple sclerosis are established. One of the main obstacles for successful gut microbiota transfer is the resilience of established intestinal microbiota to changes (13). While the resilience of “healthy” gut microbiota contributes to prevention of dysbiosis-related diseases, the resilience of already dysbiotic gut microbiota prevents successful introduction of beneficial microbes in the community. Importantly, it has recently been shown that short Antb treatment followed by short gut microbiota transfer (both treatments lasting just for two days) executed 3 days after EAE immunization protected mice from the disease (46). Such a short-lasting efficient treatment of humans would be a revolutionary therapeutic advance. Of course, the efficiency of the procedure will have to be checked in different multiple sclerosis models before translation to humans. Our unpublished data show that the same protocol is not efficient in transfer of AO rat gut microbiota transfer to DA rats.

Finally, an interesting observation regarding IL-17 and IFN-γ production in the spinal cord of Antb-treated AO rats came out, as the level of the former was increased and the level of the latter was decreased in comparison to untreated EAE Ctrls. Accordingly, beneficial effect of AO rat gut microbiota transfer into DA rats was paralleled with decrease of IL-17, but increase of IFN-γ in the spinal cord. Differential contribution of IL-17- and IFN-γ-generating cells in the CNS autoimmunity has been already acknowledged (47). Different regions of the CNS have been preferentially affected by IL-17- or IFN-γ-generating cells and as a consequence various clinical outcomes were observed (48). Although Th17 cells were dominant in brain and not spinal cord inflammation in mice, the opposite was determined in humans (48, 49). Our results suggest that rats are more similar to humans than mice in that matter. Also, it has been proposed that IFN-γ has regulatory property in the brain, but not in the spinal cord of mice (48). Still, relatively high IL-17 to IFN-γ ratio in the spinal cord seemed to be a hallmark of clinically manifested EAE in AO rats, while the opposite ratio was observed with amelioration of EAE in DA rats. Further research is needed to investigate if IFN-γ has important anti-inflammatory and neuroprotective role in rat spinal cords.

To conclude, further studies on multiple sclerosis-related gut microorganisms and the ways on successful incorporation of beneficial microbes into presumably dysbiotic intestinal microbiota community are needed before using gut microbiota transfer as a therapy for the disease. Our study contributes to the view that gut microbiota is an important factor in the CNS autoimmunity. Also, it corroborates the idea that gut microbiota modulation could be a useful approach in multiple sclerosis therapy.

## Ethics Statement

This study was carried out in accordance with the recommendations of the Directive 2010/63/EU of the European Parliament and of the Council of 22 September 2010 on the protection of animals used for scientific purposes. The protocol was approved by the Ethics committee of the Institute for Biological Research “SiniŠa Stanković,” No 04-04/15.

## Author Contributions

SS: performed main work and analyzed, interpreted, and critically revised the data; MD: performed the work related to DGGE and sequencing and analysis of the sequenced data; BJ: worked on animal model, organ isolation, cell preparation, and cytofluorimetry; NĐ: worked on animal model, organ isolation, cell preparation, and ELISA; MM: worked on animal model, rat treatment, organ isolation, and cell preparation; JĐ: performed the DGGE analysis, statistical analysis, and made part of the draft related to DGGE analysis; NG: microbiology—conception and design, supervised the work, analyzed and interpreted the data, and critically revised the manuscript; MS: conception and design related to immunology and critically revised the manuscript; ĐM: immunology—conception and design, supervised the work, analyzed and interpreted the data, drafted the work, and critically revised the manuscript. All authors finally approved the version to be published and agreed to be accountable for all aspects of the work in ensuring that questions related to the accuracy or integrity of any part of the work are appropriately investigated and resolved.

## Conflict of Interest Statement

The authors declare that the research was conducted in the absence of any commercial or financial relationships that could be construed as a potential conflict of interest.

## Abbreviations

AO, Albino Oxford; Antb, antibiotics; c.s., clinical score; CFA, complete Freund’s adjuvant; CNS, central nervous system; Ctrl, control; d.p.i., days post immunization; DA, Dark Agouti; DGGE, denaturing gradient gel electrophoresis; EAE, experimental autoimmune encephalomyelitis; FCS, fetal calf serum; GALT, gut-associated lymphoid tissue; Gmbt, gut microbiota transfer; IFN, interferon; IL, interleukin; MBP, myelin basic protein; MLN, mesenteric lymph node; MLNC, MLN cells; OTU, operational taxonomic units; PBS, phosphate buffer saline; PCR, polymerase chain reaction; PP, Peyer’s patch; PPC, PP cells; Th, helper T cells; Treg, regulatory T cells.

## References

[1] Lee YK Mazmanian SK. Has the microbiota played a critical role in the evolution of the adaptive immune system? Science (2010) 330(6012):1768–73. 10.1126/science.1195568 21205662 PMC3159383

[2] Maynard CL Elson CO Hatton RD Weaver CT. Reciprocal interactions of the intestinal microbiota and immune system. Nature (2012) 489(7415):231–41. 10.1038/nature11551 22972296 PMC4492337

[3] Shamriz O Mizrahi H Werbner M Shoenfeld Y Avni O Koren O. Microbiota at the crossroads of autoimmunity. Autoimmun Rev (2016) 15(9):859–69. 10.1016/j.autrev.2016.07.012 27392501

[4] Wekerle H. Brain autoimmunity and intestinal microbiota: 100 trillion game changers. Trends Immunol (2017) 38(7):483–97. 10.1016/j.it.2017.03.008 28601415

[5] Petermann F Korn T. Cytokines and effector T cell subsets causing autoimmune CNS disease. FEBS Lett (2011) 585(23):3747–57. 10.1016/j.febslet.2011.03.064 21477588

[6] Westall FC. Molecular mimicry revisited: gut bacteria and multiple sclerosis. J Clin Microbiol (2006) 44(6):2099–104. 10.1128/JCM.02532-05 16757604 PMC1489420

[7] Cantarel BL Waubant E Chehoud C Kuczynski J De Santis TZ Warrington J Gut microbiota in multiple sclerosis: possible influence of immunomodulators. J Investig Med (2015) 63:729–34. 10.1097/JIM.000000000000019 25775034 PMC4439263

[8] Miyake S Kim S Suda W Oshima K Nakamura M Matsuoka T Disbiosis in the gut microbiota of patients with multiple sclerosis, with a striking depletion of species belonging to clostridia XIVa and IV clusters. PLoS One (2015) 10(9):e0137429 10.1371/journal.pone.0137429 26367776 PMC4569432

[9] Jangi S Gandhi R Cox LM Li N von Glehn F Yan R Alterations of the human gut microbiome in multiple sclerosis. Nat Commun (2016) 7:12015. 10.1038/ncomms12015 27352007 PMC4931233

[10] Chen J Chia N Kalari KR Yao JZ Novotna M Soldan MM Multiple sclerosis patients have a distinct gut microbiota compared to healthy controls. Sci Rep (2016) 6:28484. 10.1038/srep28484 27346372 PMC4921909

[11] Cekanaviciute E Yoo BB Runia TF Debelius JW Singh S Nelson CA Gut bacteria from multiple sclerosis patients modulate human T cells and exacerbate symptoms in mouse models. Proc Natl Acad Sci U S A (2017) 114(40):10713–8. 10.1073/pnas.1711235114 28893978 PMC5635915

[12] Berer K Gerdes LA Cekanaviciute E Jia X Xiao L Xia Z Gut microbiota from multiple sclerosis patients enables spontaneous autoimmune encephalomyelitis in mice. Proc Natl Acad Sci U S A (2017) 114(40):10719–24. 10.1073/pnas.1711233114 28893994 PMC5635914

[13] Sommer F Anderson JM Bharti R Raes J Rosenstiel P. The resilience of the intestinal microbiota influences health and disease. Nat Rev Microbiol (2017) 15(10):630–8. 10.1038/nrmicro.2017.58 28626231

[14] Momcilović M Mostarica-Stojković M Miljković D. CXCL12 in control of neuroinflammation. Immunol Res (2012) 52(1–2):53–63. 10.1007/s12026-012-8282-x 22392052

[15] Stanisavljević S Lukić J Momčilović M Miljković M Jevtić B Kojić M Gut-associated lymphoid tissue, gut microbes and susceptibility to experimental autoimmune encephalomyelitis. Benef Microbes (2016) 7(3):363–73. 10.3920/BM2015.0159 26839070

[16] Stanisavljević S Lukić J Soković S Mihajlovic S Mostarica Stojković M Miljković D Correlation of gut microbiota composition with resistance to experimental autoimmune encephalomyelitis in rats. Front Microbiol (2016) 7:2005. 10.3389/fmicb.2016.02005 28018327 PMC5156687

[17] Lukic J Strahinic I Milenkovic M Golic N Kojic M Topisirovic L Interaction of *Lactobacillus fermentum* BGHI14 with rat colonic mucosa: implications for colitis induction. Appl Environ Microbiol (2013) 79(18):5735–44. 10.1128/AEM.01807-13 23851097 PMC3754154

[18] Heilig HGHJ Zoetendal EG Vaughan EE Marteau P Akkermans ADL de Vos WDL Molecular diversity of *Lactobacillus* spp. and other lactic acid bacteria in the human intestine by specific amplification of 16S ribosomal DNA. Appl Environ Microbiol (2002) 68(1):114–23. 10.1128/AEM.68.1.114-123.2002 11772617 PMC126540

[19] Randazzo CL Vaughan EE Caggia C. Artisanal and experimental pecorino siciliano cheese: microbial dynamics during manufacture assessed by culturing and PCR-DGGE analyses. Int J Food Microbiol (2006) 9(1–2):1–8. 10.1016/j.ijfoodmicro.2005.11.002 16616965

[20] Hanahan D. Studies on transformation of *Escherichia coli* with plasmids. J Mol Biol (1983) 166(4):557–80. 10.1016/S0022-2836(83)80284-8 6345791

[21] Miljkovic D Stosic-Grujicic S Markovic M Momcilovic M Ramic Z Maksimovic-Ivanic D Strain difference in susceptibility to experimental autoimmune encephalomyelitis between Albino Oxford and dark agouti rats correlates with disparity in production of IL-17, but not nitric oxide. J Neurosci Res (2006) 84(2):379–88. 10.1002/jnr.20883 16676327

[22] Francino MP. Early development of the gut microbiota and immune health. Pathogens (2014) 3(3):769–90. 10.3390/pathogens3030769 25438024 PMC4243441

[23] Yamamura T Da-Lin Y Satoh J Tabira T. Suppression of experimental allergic encephalomyelitis by 15-deoxyspergualin. J Neurol Sci (1987) 82(1–3):101–10. 10.1016/0022-510X(87)90010-4 3502003

[24] Matsushima S Yoshitoshi T Shichi H. Immunosuppression by gramicidin S of experimental autoimmune uveoretinitis, pinealitis and autoimmune encephalomyelitis. J Ocul Pharmacol (1990) 6(3):219–26. 10.1089/jop.1990.6.219 1705277

[25] Popovic N Schubart A Goetz BD Zhang SC Linington C Duncan ID. Inhibition of autoimmune encephalomyelitis by a tetracycline. Ann Neurol (2002) 51(2):215–23. 10.1002/ana.10092 11835378

[26] Melzer N Meuth SG Torres-Salazar D Bittner S Zozulya AL Weidenfeller C A beta-lactam antibiotic dampens excitotoxic inflammatory CNS damage in a mouse model of multiple sclerosis. PLoS One (2008) 3(9):e3149. 10.1371/journal.pone.0003149 18773080 PMC2522272

[27] Wang D Lu Z Hu L Zhang Y Hu X. Macrolide antibiotics aggravate experimental autoimmune encephalomyelitis and inhibit inducible nitric oxide synthase. Immunol Invest (2009) 38(7):602–12. 10.1080/08820130903062194 19811424

[28] Ma K Chen X Chen JC Wang Y Zhang XM Huang F Rifampicin attenuates experimental autoimmune encephalomyelitis by inhibiting pathogenic Th17 cells responses. J Neurochem (2016) 139(6):1151–62. 10.1111/jnc.13871 27774592 PMC6680363

[29] Ochoa-Repáraz J Mielcarz DW Ditrio LE Burroughs AR Foureau DM Haque-Begum S Role of gut commensal microflora in the development of experimental autoimmune encephalomyelitis. J Immunol (2009) 183(10):6041–50. 10.4049/jimmunol.0900747 19841183

[30] Ochoa-Repáraz J Mielcarz DW Haque-Begum S Kasper LH. Induction of a regulatory B cell population in experimental allergic encephalomyelitis by alteration of the gut commensal microflora. Gut Microbes (2010) 1(2):103–8. 10.4161/gmic.1.2.11515 21326918 PMC3023588

[31] Torow N Hornef MW. The neonatal window of opportunity: setting the stage for life-long host-microbial interaction and immune homeostasis. J Immunol (2017) 198(2):557–63. 10.4049/jimmunol.1601253 28069750

[32] Zanvit P Konkel JE Jiao X Kasagi S Zhang D Wu R Antibiotics in neonatal life increase murine susceptibility to experimental psoriasis. Nat Commun (2015) 6:8424. 10.1038/ncomms9424 26416167 PMC4598725

[33] Ferrer M Méndez-García C Rojo D Barbas C Moya A. Antibiotic use and microbiome function. Biochem Pharmacol (2017) 134:114–26. 10.1016/j.bcp.2016.09.007 27641814

[34] Hu Y Jin P Peng J Zhang X Wong FS Wen L. Different immunological responses to early-life antibiotic exposure affecting autoimmune diabetes development in NOD mice. J Autoimmun (2016) 72:47–56. 10.1016/j.jaut.2016.05.001 27178773 PMC4958594

[35] Mu Q Tavella VJ Kirby JL Cecere TE Chung M Lee J Antibiotics ameliorate lupus-like symptoms in mice. Sci Rep (2017) 7(1):13675. 10.1038/s41598-017-14223-0 29057975 PMC5651817

[36] Tamanai-Shacoori Z Smida I Bousarghin L Loreal O Meuric V Fong SB *Roseburia* spp.: a marker of health? Future Microbiol (2017) 2:157–70. 10.2217/fmb-2016-0130 28139139

[37] St Charles JL Bell JA Gadsden BJ Malik A Cooke H Van de Grift LK Guillain Barré syndrome is induced in non-obese diabetic (NOD) mice following *Campylobacter jejuni* infection and is exacerbated by antibiotics. J Autoimmun (2017) 77:11–38. 10.1016/j.jaut.2016.09.003 27939129

[38] Sun J Furio L Mecheri R van der Does AM Lundeberg E Saveanu L Pancreatic β-cells limit autoimmune diabetes via an immunoregulatory antimicrobial peptide expressed under the influence of the gut microbiota. Immunity (2015) 43(2):304–17. 10.1016/j.immuni.2015.07.013 26253786

[39] Fahey JR Lyons BL Olekszak HL Mourino AJ Ratiu JJ Racine JJ Antibiotic-associated manipulation of the gut microbiota and phenotypic restoration in NOD mice. Comp Med (2017) 67(4):335–43. 28830580 PMC5557205

[40] Vangay P Ward T Gerber JS Knights D. Antibiotics, pediatric dysbiosis, and disease. Cell Host Microbe (2015) 17(5):553–64. 10.1016/j.chom.2015.04.006 25974298 PMC5555213

[41] Ochoa Repáraz J Mielcarz DW Wang Y Begum-Haque S Dasgupta S Kasper DL A polysaccharide from the human commensal *Bacteroides fragilis* protects against CNS demyelinating disease. Mucosal Immunol (2010) 3(5):487–95. 10.1038/mi.2010.29 20531465

[42] Ochoa-Repáraz J Mielcarz DW Ditrio LE Burroughs AR Begum-Haque S Dasgupta S Central nervous system demyelinating disease protection by the human commensal *Bacteroides fragilis* depends on polysaccharide A expression. J Immunol (2010) 185(7):4101–8. 10.4049/jimmunol.1001443 20817872

[43] Wang Y Begum-Haque S Telesford KM Ochoa-Repáraz J Christy M Kasper EJ A commensal bacterial product elicits and modulates migratory capacity of CD39(+) CD4 T regulatory subsets in the suppression of neuroinflammation. Gut Microbes (2014) 5(4):552–61. 10.4161/gmic.29797 25006655

[44] Wang Y Telesford KM Ochoa-Repáraz J Haque-Begum S Christy M Kasper EJ An intestinal commensal symbiosis factor controls neuroinflammation via TLR2-mediated CD39 signalling. Nat Commun (2014) 5:4432. 10.1038/ncomms5432 25043484 PMC4118494

[45] Evrensel A Ceylan ME. Fecal microbiota transplantation and its usage in neuropsychiatric disorders. Clin Psychopharmacol Neurosci (2016) 14(3):231–7. 10.9758/cpn.2016.14.3.231 27489376 PMC4977816

[46] Chitrala KN Guan H Singh NP Busbee B Gandy A Mehrpouya-Bahrami P CD44 deletion leading to attenuation of experimental autoimmune encephalomyelitis results from alterations in gut microbiome in mice. Eur J Immunol (2017) 47(7):1188–99. 10.1002/eji.201646792 28543188 PMC5704912

[47] Domingues HS Mues M Lassmann H Wekerle H Krishnamoorthy G. Functional and pathogenic differences of Th1 and Th17 cells in experimental autoimmune encephalomyelitis. PLoS One (2010) 5(11):e15531. 10.1371/journal.pone.0015531 21209700 PMC3000428

[48] Pierson E Simmons SB Castelli L Goverman JM. Mechanisms regulating regional localization of inflammation during CNS autoimmunity. Immunol Rev (2012) 248(1):205–15. 10.1111/j.1600-065X.2012.01126.x 22725963 PMC3678350

[49] Johnson MC Pierson ER Spieker AJ Nielsen AS Posso S Kita M Distinct T cell signatures define subsets of patients with multiple sclerosis. Neurol Neuroimmunol Neuroinflamm (2016) 3(5):e278. 10.1212/NXI.0000000000000278 27606354 PMC4996538

